# Supplementary material for: Centromere-proximal suppression of meiotic crossovers in Drosophila is robust to changes in centromere number, repetitive DNA content, and centromere-clustering
Source: Genetics. 2023 Dec 27;226(3):iyad216. doi: 10.1093/genetics/iyad216 (PMC10917511; doi:10.1093/genetics/iyad216)
Supplement: iyad216_Supplementary_Data [file iyad216_supplementary_data.pdf]

Supplemental Material

for

Centromere-Proximal Suppression of Meiotic Crossovers in *Drosophila* is Robust to Changes in Centromere Number, Repetitive DNA Content, and Centromere-Clustering

Nila M. Pazhayam, Leah K. Frazier, Jeff Sekelsky

Figure S1: Mitotic spreads with CENP-C foci in compound chromosomes

Table S1: Datasets for chromosome *2* crossovers

Table S2: Datasets for chromosome *3* crossovers

Table S3: Counts of CENP-C foci

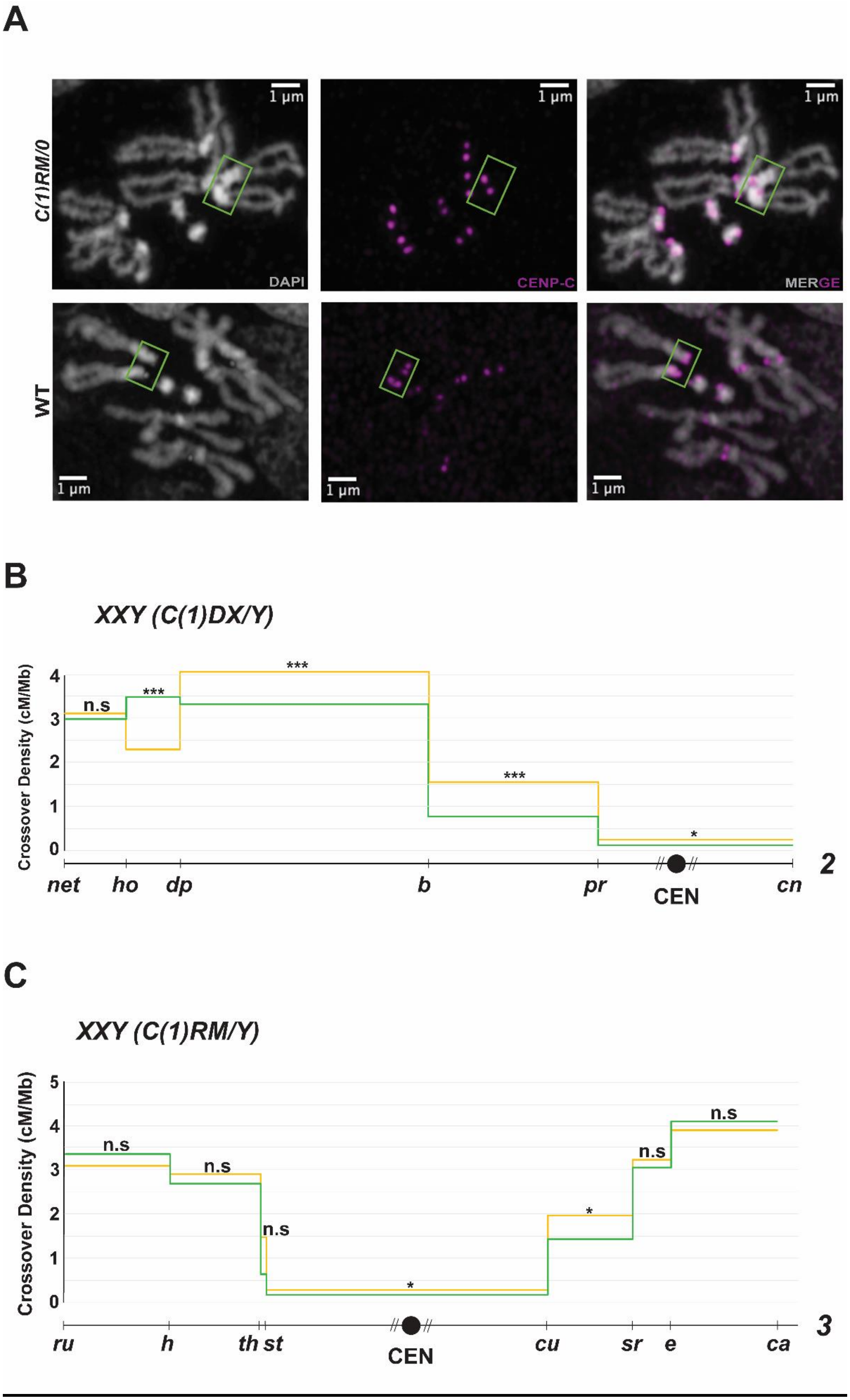
A
C(1)RM/0
WT
1 μm
DAPI
CENP-C
MERGE
B
XXY (C(1)DX/Y)
Crossover Density (cM/Mb)
n.s
***
***
***
*
net
ho
dp
b
pr
CEN
cn
2
C
XXY (C(1)RM/Y)
Crossover Density (cM/Mb)
n.s
n.s
n.s
*
*
n.s
n.s
ru
h
th st
CEN
cu
sr
e
ca
3

<u>Figure S1.</u> **(A)** Mitotic chromosome spreads (grey) with CENP-C foci (magenta) from *C(1)/0* larvae (top panel) and wild type larvae (bottom panel). Green boxes highlight chromosome *X* (larger, V-shaped structure) in both genotypes. **(B)** CO distribution along chromosome *2* in *C(1)DX/Y* flies (yellow line, *n*=2702) and wild type flies (green line, *n*= 4104). CO density in cM/Mb is indicated on the Y-axis, relative physical distances between markers used to score COs are indicated on the X-axis. The black circle represents the centromere, with dashed lines around it representing pericentromeric repetitive sequence that remains unassembled. Statistical significance in each interval was calculated using two-tailed Fischer's exact test between the difference in total CO versus NCO numbers in mutants from wild-type flies (ns $p>0.0083$, *$p<0.0083$, **$p<0.0017$, ***$p<0.00017$ after correction for multiple comparisons). Complete dataset can be found in Table S1. **(C)** CO distribution along chromosome *3* in *C(1)RM/Y* flies (yellow line, *n*=1854) and wild type flies (green line, *n*= 1728). CO density in cM/Mb is indicated on the Y-axis, relative physical distances between markers used to score COs are indicated on the X-axis. The black circle represents the centromere, with dashed lines around it representing pericentromeric repetitive sequence that remains unassembled. Statistical significance in each interval was calculated using two-tailed Fischer's exact test between the difference in total CO versus NCO numbers in mutants from wild-type flies (ns $p>0.0125$, *$p<0.0125$, **$p<0.0025$, ***$p<0.00025$ after correction for multiple comparisons). Complete dataset can be found in Tables S2.

Table S1. Dataset used to make the chromosome *2* graphs in Fig. 1, 2, 4, and S1. The number of parental, single crossover (SCO), double crossover (DCO), and triple crossover (TCO) flies in each chromosome constitution are shown.

	Progeny	Genotype Being Assayed						
		WT	*C(1)RM/0* ; *C(4)RM/0*	*C(1)RM/Y* ; *C(4)RM/+*	*C(1)RM/Y*	*C(4)RM/+*	*D1*^{LL03310}/ *Df(3R)BSC*^{666}	*C(1)DX/Y*
	Parental	2363	2700	2380	3144	1779	4141	1312
SCO	1 (*net-ho*)	243	213	232	128	27	160	132
SCO	2 (*ho-dp*)	242	388	208	108	61	268	80
SCO	3 (*dp-b*)	1178	1559	1213	621	895	1505	909
SCO	4 (*b-pr*)	165	295	239	80	89	396	199
SCO	5 (*pr-cn*)	38	61	81	50	34	62	41
DCO	1 and 2	6	2	11	1	2	20	3
DCO	1 and 3	12	21	19	7	1	3	23
DCO	1 and 4	7	24	12	7	0	8	12
DCO	1 and 5	2	9	4	1	0	0	6
DCO	2 and 3	15	94	15	2	4	5	9
DCO	2 and 4	11	27	8	5	2	6	12
DCO	2 and 5	1	8	2	1	1	3	3
DCO	3 and 4	17	69	18	6	15	26	37
DCO	3 and 5	18	22	26	4	12	21	19
DCO	4 and 5	2	4	1	3	2	3	4
TCO	1, 2, and 3	11	8	39	0	0	7	18
TCO	1, 2, and 4	0	0	4	0	0	0	0
TCO	1, 2, and 5	0	0	4	0	0	0	0
TCO	1, 3, and 4	0	1	0	0	0	0	0
TCO	1, 3, and 5	0	2	0	1	0	0	1
TCO	1, 4, and 5	0	0	0	0	0	0	0
TCO	2, 3, and 4	0	8	0	0	0	0	0
TCO	2, 3, and 5	0	2	0	0	0	0	0
TCO	2, 4, and 5	0	0	0	0	0	0	0
TCO	3, 4, and 5	0	3	2	0	0	1	0
	Total *n*	4104	5311	4434	3787	2924	6399	2702

Table S2. Table showing the complete dataset used to make the chromosome *3* graphs in Fig. 2, 3, 4, and S1. The number of parental, single crossover (SCO), double crossover (DCO), and triple crossover (TCO) flies in each mutant are shown.

	**Progeny**	**Genotype Being Assayed**				
		WT	*C(1)RM/Y* ; *C(4)RM/+*	*C(1)RM/Y*	*Df(2R)M41A10*	*Prod^{K}/+*
	Parental	413	206	446	492	1472
SCO	1 (*ru-h*)	211	96	205	160	584
	2 (*h-th*)	171	92	137	199	450
	3 (*th-st*)	0	4	6	4	7
	4 (*st-cu*)	29	12	49	38	62
	5 (*cu-sr*)	73	58	97	106	254
	6 (*sr-e*)	75	33	76	68	222
	7 (*e-ca*)	269	153	279	265	943
DCO	1 and 2	8	13	23	20	37
	1 and 3	1	0	2	0	2
	1 and 4	12	4	15	8	23
	1 and 5	29	14	43	46	94
	1 and 6	42	16	31	39	91
	1 and 7	158	67	103	74	347
	2 and 3	0	0	0	0	0
	2 and 4	4	1	7	4	9
	2 and 5	19	24	39	34	64
	2 and 6	19	7	27	27	78
	2 and 7	87	48	107	97	257
	3 and 4	0	0	0	0	0
	3 and 5	1	0	2	0	0
	3 and 6	0	0	1	0	2
	3 and 7	1	1	0	2	8
	4 and 5	3	4	6	2	13
	4 and 6	2	1	5	3	5
	4 and 7	16	8	13	19	39
	5 and 6	3	0	2	1	0
	5 and 7	21	18	26	19	70
	6 and 7	12	4	14	5	39

TCO	1, 2, and 3	0	0	0	0	0
	1, 2, and 4	0	0	1	0	0
	1, 2, and 5	2	4	2	1	0
	1, 2, and 6	1	1	3	0	4
	1, 2, and 7	10	9	17	4	10
	1, 3, and 4	1	0	0	0	0
	1, 3, and 5	0	1	0	0	0
	1, 3, and 6	0	0	0	0	1
	1, 3, and 7	0	2	0	0	2
	1, 4, and 5	0	2	1	2	4
	1, 4, and 6	0	0	4	2	1
	1, 4, and 7	2	2	9	8	12
	1, 5, and 6	1	0	1	0	0
	1, 5, and 7	11	6	13	5	24
	1, 6, and 7	3	1	12	0	8
	2, 3, and 4	0	0	0	0	0
	2, 3, and 5	0	0	0	0	0
	2, 3, and 6	0	0	0	0	0
	2, 3, and 7	0	0	0	0	0
	2, 4, and 5	0	0	1	1	1
	2, 4, and 6	0	0	1	0	0
	2, 4, and 7	3	2	8	2	3
	2, 5, and 6	0	0	3	1	1
	2, 5, and 7	10	6	11	8	20
	2, 6, and 7	4	0	4	5	10
	3, 4, and 5	0	0	0	0	0
	3, 4, and 6	0	1	1	0	0
	3, 4, and 7	0	0	0	0	0
	3, 5, and 6	0	0	0	0	0
	3, 5, and 7	0	0	0	0	0
	3, 6, and 7	1	0	0	0	0
	4, 5, and 6	0	0	0	0	0
	4, 5, and 7	0	1	1	1	5
	4, 6, and 7	0	0	0	0	0
	5, 6, and 7	0	0	0	1	1
	Total *n*	1728	922	1854	1773	5320

<u>Table S3.</u> Number of CENP-C foci in meiotic cells of wild-type, *prod*^{K} */ +*, and *D1/ Df* females.

A. Wild type

	CENP-C foci in C(3)G positive cells that co-localized with DAPI dense regions						
	1	2	3	4	5	6	Total
image 1	2	5	5				12
image 2	1	2	1				4
image 4	1		2				3
image 5	1	2	3				6
image 8	4	4	2	2			12
image 9	3	6	1	1			11
image 11		1	2	2			5
image 12	1	1	1		1		4
image 13	1	3	2				6
image 14	2	4	6	1			13
image 16	4	7	1				12
image 17	5	4	5	1			15
image 18	1	5	5				11
total	26	44	36	7	1		114
total clustered	106						
total declustered	8						
% declustering	7.02						

B. *Prod*^{K}*/+*

	CENP-C foci in C(3)G positive cells that co-localized with DAPI dense regions						
	1	2	3	4	5	6	Total
image 1	2	2	1				5
image 2	3	6	1				10
image 3	2	6					8
image 4	3	5	2				10
image 5	6	8	1				15
image 7	5	6					11
image 8	2	7	1				10
image 9	4	6	4				14
image 10	2	10	2	1			15
image 11	6	14	3	1			24
image 12		3					3
total	35	73	15	2	0		125
total clustered	123						
total declustered	2						
% declustering	1.6						

C. *D1/Df*

	CENP-C foci in C(3)G positive cells that co-localized with DAPI dense regions						
	1	2	3	4	5	6	
image 1	3	8	11	12	2	3	39
image 2	3	3	6	4			16
image 3		6	5	8	1		20
image 4 I+II		3	7	1			11
image 5 I	1		8	1			10
image 5 II			2	1			3
image 6	4	9	8	2			23
total	11	29	47	29	3	3	122
total clustered	87						
total declustered	35						
% declustering	28.7						
